# Inhibition of Endoplasmic Reticulum Stress and Atherosclerosis by 2-Aminopurine in Apolipoprotein E-Deficient Mice

**DOI:** 10.1155/2013/847310

**Published:** 2013-07-31

**Authors:** Lichun Zhou, Dezhi Yang, Dong Fang Wu, Zhong Mao Guo, Emmanuel Okoro, Hong Yang

**Affiliations:** ^1^Department of Physiology, Meharry Medical College, Nashville, TN 37208, USA; ^2^The Central Hospital of Wuhan, Wuhan 430014, China; ^3^Zhongnan Hospital of Wuhan University, Wuhan 430071, China

## Abstract

We previously reported that the apolipoprotein (apo) B48-carrying lipoproteins obtained from apoE knockout (*apoE* ^*−*/*−*^) mice, so called E^−^/B48 lipoproteins, transformed mouse macrophages into foam cells and enhanced the phosphorylation of eukaryotic translation initiation factor 2**α** (eIF-2**α**). Furthermore, the eIF-2**α** phosphorylation inhibitor, 2-aminopurine (2-AP), attenuated E^−^/B48 lipoprotein-induced foam cell formation. The present report studied the effect of 2-AP on atherosclerosis in *apoE* ^*−*/*−*^ mice. Our results showed that the level of food intake, bodyweight, plasma cholesterol, and triglycerides was comparable in *apoE* ^*−*/*−*^ mice treated with or without 2-AP. However, the mean size of atherosclerotic lesions in the aorta sinus as well as the surface area of the entire aorta of 2-AP-treated *apoE* ^*−*/*−*^ mice were reduced by about 55% and 39%, respectively, compared to samples from untreated control *apoE* ^*−*/*−*^ mice. In addition, the 2-AP-treated *apoE* ^*−*/*−*^ mice showed a significant decrease in glucose-regulated protein 78 (GRP78) and phosphorylated eIF-2**α** in their aortic samples as compared to levels in untreated control *apoE* ^*−*/*−*^ mice. These observations suggest that endoplasmic reticulum stress is a causal mechanism for the development of atherosclerosis in *apoE* ^*−*/*−*^ mice and that therapeutic strategies can be developed for using eIF-2**α** phosphorylation inhibitors, such as 2-AP, to prevent or treat atherosclerosis.

## 1. Introduction

The endoplasmic reticulum (ER) fulfills multiple cellular functions. Once ER functions are perturbed by various pathological conditions, unfolded or misfolded proteins accumulate in the ER lumen, resulting in ER stress characterized by increasing ER molecular chaperones and diminishing global protein synthesis [1]. Activation of the signaling network in response to ER stress is known as unfolded protein response (UPR). There are three distinct UPR signaling pathways triggered in response to ER stress, which are mediated by (1) RNA-dependent protein kinase-like endoplasmic reticulum kinase (PERK), (2) activating transcription factor 6 (ATF6), and (3) inositol-requiring enzyme 1 (IRE1), respectively, [2, 3]. Under physiological conditions, PERK, ATF6, and IRE1 are associated with the abundant luminal chaperone Bip (also known as glucose-regulated protein 78, GRP78). This interaction keeps PERK, ATF6, and IRE1 in an inactive state. When the ER is overloaded with newly synthesized proteins or is stimulated by agents that cause unfolded proteins to accumulate, GRP78/Bip preferentially associates with the unfolded proteins, releasing PERK, ATF6, and IRE1 to activate downstream signaling molecules. In the PERK-mediated pathway, PERKs released from the GRP78 dimerize are autophosphorylated and manifest increased catalytic activity. Activated PERK phosphorylates eIF-2*α*. The phosphorylated eIF-2*α* subsequently inhibits global protein synthesis, preventing further influx of nascent proteins into an already saturated ER lumen. Paradoxically, eIF-2*α* phosphorylation enhances the translation of activating transcription factor-4 (ATF4) and subsequently upregulates the expression of ATF4-target genes, including several protein chaperones [4]. Recent studies also demonstrated that eIF-2*α* phosphorylation upregulates ATF6 expression and enhances ATF6 activity and therefore increases the expression of its target genes [5]. 

Although UPR signaling pathways are activated classically by unfolded ER proteins, increasing evidence demonstrates that lipids/lipoproteins can also trigger UPR. For instance, a prior study reported that accumulation of free cholesterol in the ER was able to activate UPR signaling pathways in mouse peritoneal macrophages [6]. Also, studies from our laboratory demonstrated that treatment of mouse macrophages with lipoproteins carrying apolipoprotein (apo) B48 but lacking apoE (E^−^/B48) induced foam cell formation, and enhanced phosphorylation of PERK and eIF-2*α*, increased expression of ATF4 and several molecular chaperons and reduced global protein synthesis [7, 8]. Furthermore, we observed that overexpression of dominant-negative mutants of PERK or eIF-2*α*, or treatment with the eIF-2*α* inhibitor, 2-aminopurine (2-AP), suppressed foam cell formation induced by E^−^/B48 lipoproteins [7, 8]. These findings suggest that activation of the PERK-eIF2*α* signaling pathway is an underlying mechanism by which E^−^/B48 lipoproteins induce foam cell formation. Such a transformation of macrophages into a foam cells is an early step of atherogenesis.

Individuals with defective isoforms of apoE, such as apoE4, develop postprandial hypercholesterolemia and atherosclerosis [9]. Similarly, apoE knockout (a*poE* ^−/−^) mice manifest an elevated plasma cholesterol and develop atherosclerosis in a manner that resembles the human disease [10, 11]. The hypercholesterolemia in *apoE* ^−/−^ mice results mainly from the increased levels of E^−^/B48 lipoproteins. It is interesting to note that atherosclerotic lesions in the aorta sinus of *apoE* ^−/−^ mice show UPR, as reflected by increased PERK phosphorylation in the lesions [12, 13]. Data from the present report demonstrated that treatment of *apoE* ^−/−^ mice with the eIF-2*α* inhibitor 2-AP reduced atherosclerotic lesions in their aortas compared to lesions observed in control mice. Taken together, these findings suggest that activation of eIF-2*α* signaling pathway is an underlying mechanism for the development of atherosclerosis in *apoE* ^−/−^ mice and that inhibition of this UPR pathway might provide a therapy strategy for treatment of atherosclerosis.

## 2. Materials and Methods

### 2.1. Animals and 2-AP Treatment

 Male *apoE* ^−/−^ mice were obtained from Jackson Laboratory (Bar Harbor, ME). These mice were generated using embryonic stem cells from the 129 strain of mice [10] and were crossbred to C57BL/6 for over 10 generations. They were maintained under barrier conditions in a temperature-controlled environment and fed with a mouse chow containing approximately 5% fat and 19% protein by weight (Harlan Teklad, Madison, WI). At 6 weeks of age, these mice were randomly assigned to one of two groups. One group of the mice was gavage-fed 2-AP at a dose of 200 mg/kg body weight (BW) in 200 *μ*L water once every other day, and another group of mice was fed with same volume of water as a control. Food intake and growth were monitored by weighing the food and animals weekly. After 24 weeks of treatment, mice were fasted overnight and anesthetized with ketamine hydrochloride (80 mg/kg BW) and xylazine hydrochloride (16 mg/kg BW). Approximately 0.5 mL of blood was collected from the posterior vena cava of each mouse. Thereafter, a 23-gauge needle was inserted into the left ventricle and 4% paraformaldehyde was delivered into the animal at pressure of 80 mm Hg. A small incision was made into the liver to allow efflux of blood and fixative. After fixation, the heart and the proximal aorta were removed from the body and stored in 4% paraformaldehyde at 4°C before sectioning. 

### 2.2. Quantification of Atherosclerotic Lesions in the Mouse Aorta

The aorta was cut at a distance of 2 mm from the heart. The distal aorta (2 mm from the heart to the iliac bifurcation) was opened longitudinally using microscissors and pinned flat on a black wax surface in a dissecting pan under a dissecting microscope (SMZ1000, Nikon Instruments Inc., Melville, NY), as described previously [11]. This *en face* preparation was fixed overnight and stained with Oil-Red-O. The photo-image of the aorta was captured with a CoolSNAP digital camera (Nikon Instruments) mounted on the SMZ1000 dissecting microscope. The atherosclerotic lesion area and the total aortic area were measured using a MetaMorph imaging system (Nikon Instruments). 

The proximal aorta attached to the heart was used to prepare cross-sections, as described previously [11]. Briefly, the heart was sectioned transversely immediately below and parallel to a plane formed by the line between atrial leaflets. The lower portion of the heart was discarded. The portion of the heart with the attached aorta was embedded either in OCT or in paraffin, and sectioned from the attached aorta towards the root of the aorta where the aorta valves were attached. Sections (5–8 *μ*m) were cut from the site where the aorta valve cups appear at the aorta root. Every other section was collected onto a set of microscope slides. The paraffin sections were used for immunostaining as described below. The frozen sections were stained with Oil-Red-O. The slides were viewed using a microscope (E600, Nikon Instruments Inc., Melville, NY) equipped with a Cool Snaps color digital camera and a MetaMorph computer image acquisition system. The average area (*μ*m^2^) and morphological features (foam cell deposits, cholesterol clefts, lipid cores and fibrous-caps) of the lesions in 16 sections were determined for each mouse.

### 2.3. Immunohistochemical Staining

Immunostaining was performed with the use of the VECTASTAIN ABC System (Vector Laboratories, Inc., Burlingame, CA). The paraffin sections prepared as described above, was deparaffinized using xylene. The endogenous peroxidase activity was blocked with 5% hydrogen peroxide for 10 min. After blocking with 5% normal goat or rabbit serum, the sections were incubated with primary antibodies against GRP78 or phosphorylated eIF-2*α* (Abcam Inc., Cambridge, MA). After treatment with a secondary antibody produced from goats or rabbits, sections were stained with diaminobenzidine or Nova red substrates and counterstained with hematoxylin. The immunostaining of the aortic sections were viewed using a microscope equipped with an HQ2 CoolSNAP high-resolution camera (Nikon) and the MegaMorth computer image acquisition system. The images were viewed with a Nikon TE2000 fluorescence microscope. 

### 2.4. Plasma Lipid Analysis

The levels of plasma cholesterol and triglycerides were measured by spectrophotometric quantification using reagents obtained from Sigma Chemical Co. (St. Louis, MO). For measuring cholesterol, the mixture of plasma and cholesterol-reaction reagent was incubated at 37°C for 30 min, and the absorbance was read at 530 nm with a Dynex microplate reader (Thermo Labsystems, Franklin, MA). For measuring triglycerides, the mixture of plasma and triglyceride-reaction reagent was incubated at 37°C for 10 min, and the absorbance was read at 530 nm. Plasma concentrations of cholesterol and triglycerides were determined based on the absorbance obtained by incubation of the cholesterol and triglyceride standards provided by Sigma. 

 For determination of the cholesterol level in various lipoproteins, a 100 *μ*L plasma sample obtained from individual mice was fractionated using a fast performance liquid chromatography (FPLC) (Äkta FPLC 900, Amersham Biosciences, Piscataway, NJ) in a buffer containing 0.15 M NaCl, 0.01 M Na_2_HPO_4_, 0.1 mM EDTA, pH 7.5, at a flow rate of 0.5 mL/min. Forty fractions (0.5 mL/fraction) were collected. It had already been established that fractions 14–17 contain very low-density lipoprotein (VLDL) and chylomicrons, fractions 18–25 contain LDL, and fractions 26–40 contain high-density lipoprotein (HDL) [14, 15]. The cholesterol content in various lipoproteins was calculated from the concentration in the FPLC fractions [14, 15]. 

### 2.5. Western Blot Analyses

The antibodies against phosphorylated eIF-2*α*, GRP78 and lysosomal acid lipase (LAL) were obtained from Abcam Inc. (Cambridge, MA), while the antibodies against eIF-2*α* and *β*-actin were obtained from Santa Cruz Biotechnology Inc. (Santa Cruz, CA). Aortas from two mice were pooled, homogenized in 20 mM Tris-Cl and centrifuged at 14,000 rpm for 10 min at 4°C. Supernatants containing 15 *μ*g protein were separated by sodium dodecyl sulfate-polyacrylamide gel electrophoresis on 10% gels and transferred to polyvinylidene difluoride membranes. Membranes were blocked with 5% fat-free milk in TBS-T (2.5 mM Tris, 15 mM NaCl, 0.01% Tween 20; pH 7.6) and then consequently incubated with primary antibodies against indicated proteins and horseradish peroxidase-conjugated secondary antibodies, as previously described [16]. Immunoreactive bands were visualized using ECL-plus chemiluminescence reagent (GE Healthcare Healthcare-Amersham) and analyzed with a GS-700 Imaging Densitometer (Bio-Rad, Hercules, CA). 

### 2.6. Statistical Analysis

The data are reported as mean ± standard error of the mean. The differences between 2-AP treatment and control groups were analyzed by Student's *t*-test. Differences were considered significant at a *P* value less than 0.05.

## 3. Results

### 3.1. Effect of 2-AP on Body Weight and Food Intake

The pharmacological properties of 2-AP, such as effective dose 50 (ED50) and effective half-life (*t*1/2), have not been tested *in vivo*. The Material Safety Data Sheet produced by InvivoGen (San Diego, CA) indicates that the lethal dose 50 (LD50) of 2-AP via oral administration and intraperitoneal injection of rats is 723 and 270 mg/kg body weight (BW), respectively. A preliminary study in our laboratory demonstrated that gavage feeding of 2-AP to* apoE* ^−/−^ mice at doses greater than 400 mg/kg BW once every other day reduced food intake and resulted in death (data not show). In the present study, we observed that feeding 2-AP at a dose of 200 mg/kg BW once every other day did not reduce food intake and body weight, and did not increase mortality within 24 weeks as compared to untreated control *apoE* ^−/−^ mice (Table 1). Specifically, the average of food intake of *apoE* ^−/−^ mice at 24–30 weeks of age with or without 2-AP treatment was 0.24 ± 0.03 and 0.25 ± 0.07 g chow/g BW/day, respectively. The body weight of these mice at 30 weeks of age was about 27 g. No significant difference was observed between the 2-AP treated and the untreated control mice.

### 3.2. 2-AP Reduces Atherosclerosis but Does Not Affect Plasma Lipids in ApoE^−/−^ Mice

**Figure 1:**
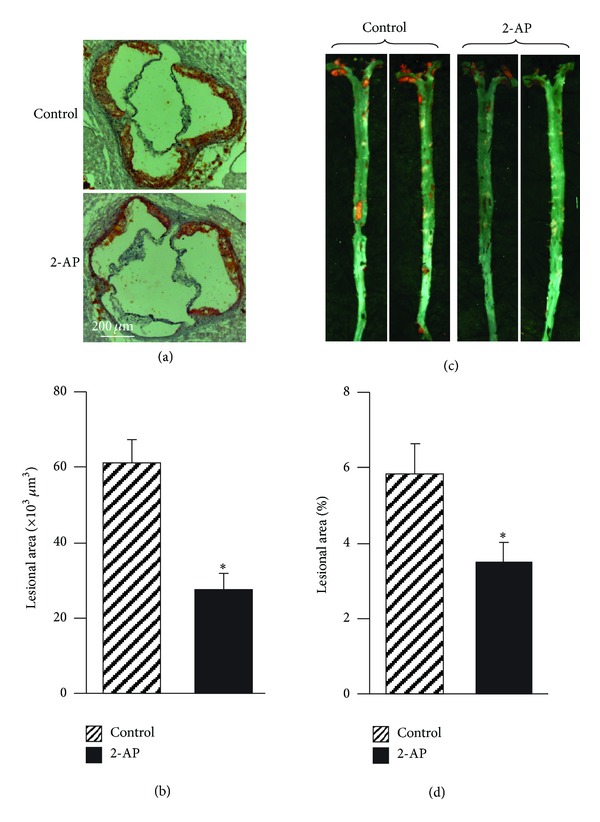
The effect of 2-AP on atherosclerosis. Male *apoE* ^−/−^ mice at 6 weeks of age were gavage-fed 200 *μ*L water (control) or 200 mg/kg BW of 2-AP once every other day for 24 weeks. Heart-aorta samples were collected. (a) Frozen sections were cut from the mouse aortic sinus and stained with Oil-Red-O. Magnification = 100. (b) The average area (*μ*m^2^) of the lesions in sections was determined in mice given 2-AP or water as a vehicle control. (c) Aortas from the aortic root to the iliac bifurcation were collected and opened longitudinally. The *en face* preparations were fixed and stained with Oil-Red-O. Magnification + 0.5x. (d) The atherosclerotic lesion area and the total aortic area were measured. Data are expressed as the percentage of the aortic surface area covered by atherosclerotic lesions, after determining the atherosclerotic lesion area and the total aortic area. Values are the mean ± SEM of 12 mice. *P < 0.05 as compared to untreated control mice.

We previously reported that 2-AP inhibited E^−^/B48 lipoprotein-induced transformation of macrophages into foam cells. The present study examined the effect of 2-AP on atherosclerosis in *apoE* ^−/−^ mice. Figures 1(a) and 1(b) show examples of cross-sections and en face preparations of aortas obtained from apoE^−/−^ mice treated with or without 2-AP. Data in Figures 1(c) and 1(d) show that the mean size of atherosclerotic lesions in the aorta sinus and the surface area of the entire aorta of 2-AP-treated apoE^−/−^ mice were reduced by about 55% and 39%, respectively, compared to those in the untreated control apoE^−/−^ mice. Consistent with the smaller lesion size, the advanced lesions were reduced markedly in 2-AP-treated mice. For instance, the number of lipid cores (acellular areas) in the aortic sinus in the 2-AP-treated and untreated control apoE^−/−^ mice were 1.2 ± 0.6 and 0.4 ± 0.1/section (*P* < 0.05), respectively. These results, together with our previous findings that 2-AP inhibits E^−^/B48 lipoprotein-induced foam cell formation [7], suggest that 2-AP is able to inhibit atherogenesis *in vitro* and *in vivo*.

**Figure 2:**
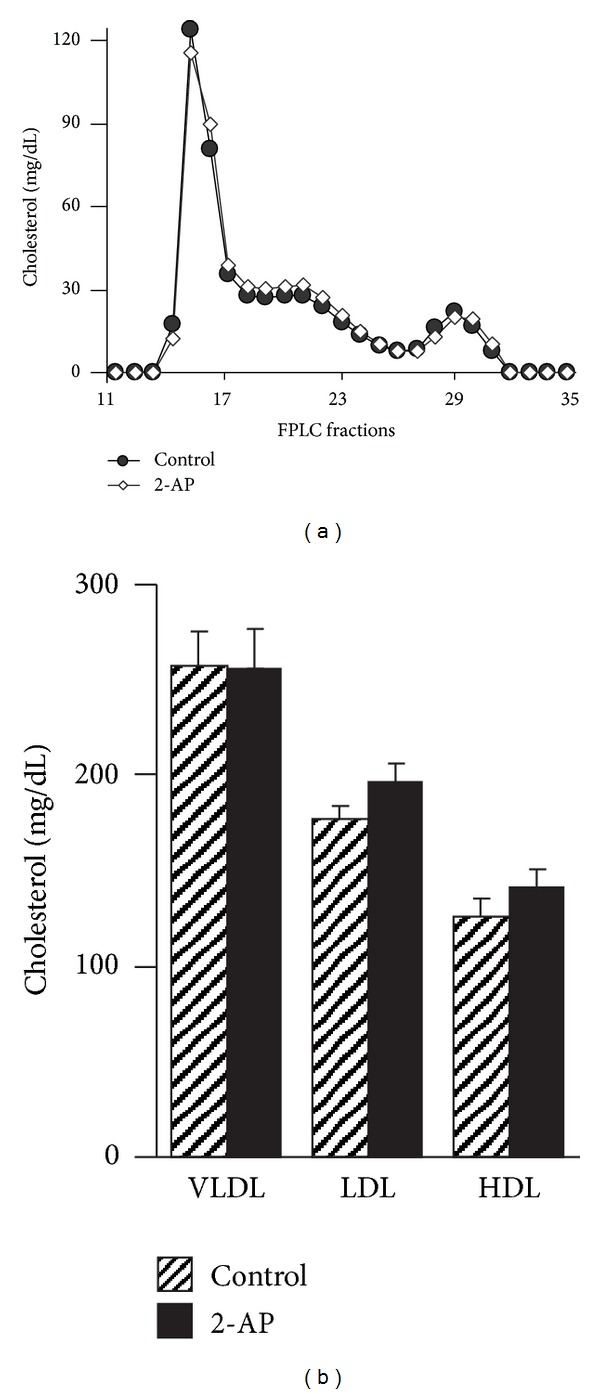
Distribution of cholesterol among plasma lipoprotein fractions. Plasma obtained from 2-AP-treated *apoE* ^−/−^ mice and untreated control mice was fractionated with a FPLC system. (a) Representative FPLC cholesterol profiles for each group of mice. (b) Cholesterol levels in plasma lipoprotein fractions obtained from control and 2-AP treated* apoE* ^−/−^ mice.

An increase in plasma cholesterol and triglycerides has been suggested to be a risk factor for atherosclerosis [17]. To determine whether the reduction in atherosclerotic lesions induced by 2-AP treatment was due to a change in plasma cholesterol and triglycerides, we measured the levels of plasma cholesterol and triglycerides in *apoE* ^−/−^ mice with or without 2-AP treatment. As the data in Table 1 show that the average plasma concentrations of total cholesterol and triglycerides were 513 and 146, respectively, in *apoE* ^−/−^ mice without 2-AP treatment and that 2-AP treatment did not significantly alter these plasma lipid levels. To investigate whether 2-AP feeding affected the distribution of cholesterol among the lipoproteins, we fractionated mouse plasma with an FPLC system (Figure 2(a)). As the data in Figure 2(b) show, the plasma cholesterol levels in VLDL, LDL, and HDL fractions were comparable in 2-AP-treated and untreated control mice. These results suggest that the reduced atherosclerotic lesions in 2-AP treated mice were not due to altered levels of plasma lipids.

### 3.3. 2-AP Reduces ER Stress in the Aortas of ApoE^−/−^ Mice

**Figure 3:**
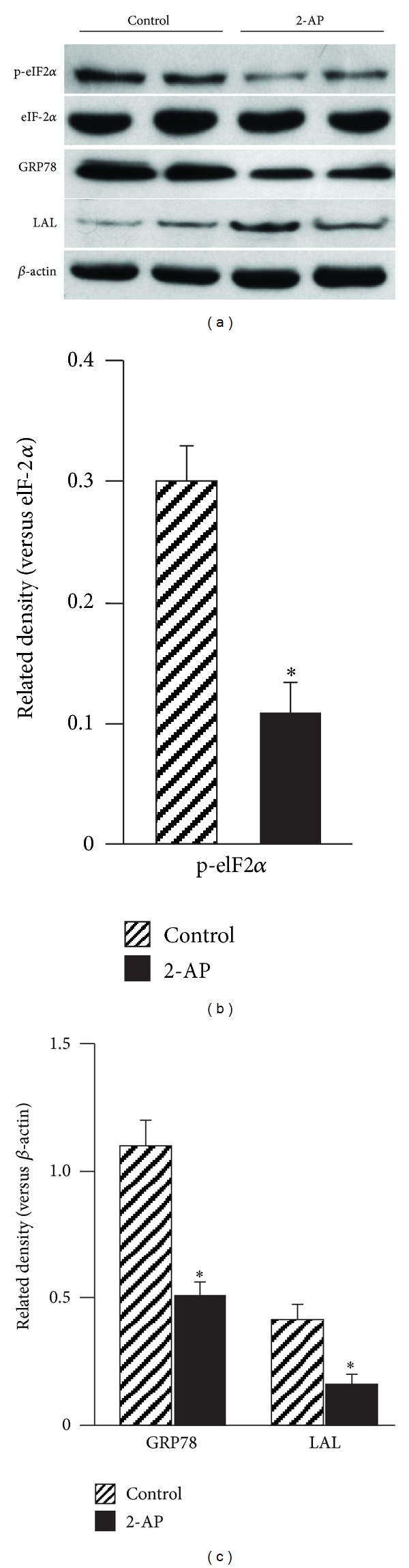
The effect of 2-AP on protein expression in the aorta. Distal regions of aortas from control and 2-AP treated *apoE* ^−/−^ mice were minced and homogenized. Protein levels in the aorta extract were determined with western blot analysis using antibodies against eIF-2*α* and phosphorylated eIF-2*α* (p-eIF2*α*), GRP78, LAL (liposomal acid lipase), and *β*-actin. The level of p-eIF2*α* was expressed relative to the eIF-2*α* immunoblot intensity. The levels of the other proteins were expressed relative to *β*-actin. Data are expressed as the mean ± SEM of three independent experiments. *P < 0.05 as compared to untreated control mice.

We previously reported that transformation of macrophages into foam cells by E^−^/B48 lipoproteins was associated with ER stress, as reflected by increased phosphorylation of PERK and eIF-2*α*. Further that 2-AP inhibited E^−^/B48 lipoprotein-induced foam cell formation and reduced PERK and eIF-2*α* phosphorylation. The present report studied the effect of 2-AP on the expression level of total and phosphorylated eIF-2*α* in the aorta of *apoE* ^−/−^ mice. Data in Figure 3(a) show that the level of total eIF-2*α* was comparable in aortas obtained from *apoE* ^−/−^ mice treated with or without 2-AP treatment. In contrast, the level of phosphorylated eIF-2*α* in the aorta was about 64% lower in 2-AP-treated *apoE* ^−/−^ mice than in untreated control mice (Figure 3(b)). The data in Figure 3(c) demonstrate that the protein level of chaperon GRP78 was reduced by ~53% in 2-AP-treated *apoE* ^−/−^ mice as compared to the untreated control littermates.

We previously reported that intralysosomal accumulation of lipids/lipoproteins induced by E^−^/B48 lipoproteins was associated with decreased lysosomal hydrolase LAL, and that 2-AP treatment restored E^−^/B48 lipoprotein-reduced expression of LAL. Therefore, the present report compared the protein levels of LAL in the aorta of *apoE* ^−/−^ mice with or without 2-AP treatment. Data in Figures 3(a) and 3(c) show that the protein level of aortic LAL was ~2.6 fold higher in 2-AP-treated *apoE* ^−/−^ mice than in their untreated littermates.

**Figure 4:**
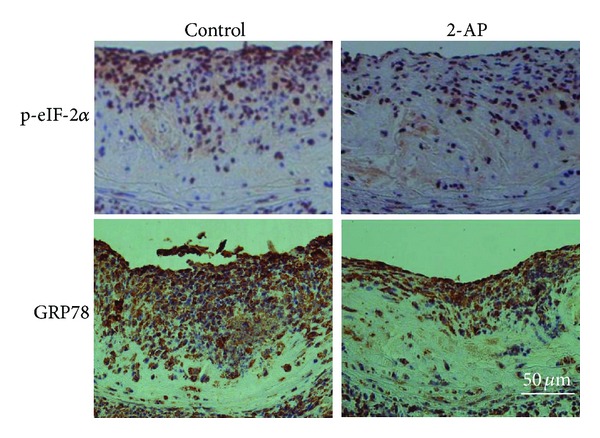
Representative immunochemical staining sections of aorta sinus. The heart-aorta samples were collected from *apoE* ^−/−^ mice fed 2-AP or equal volume of water (control). Paraffin sections were cut from the aorta sinus and immunostained with antibodies against p-eIF-2*α* or GRP78. Magnification = 200x.

 Having demonstrated the inhibitory activity of 2-AP on ER stress in the aorta, we then compared the expression of GRP78 and phosphorylated eIF-2*α* in the lesional area in the aorta sinus. As the representative immunostaining sections in Figure 4 show, the atherosclerotic lesions, especially the areas close to the plaque surface, accumulated cells positively stained by antibodies against GRP78 or phosphorylated eIF-2*α*. The number of positively stained cells in 2-AP-treated *apoE* ^−/−^ mice was much less than those in untreated control mice. These results are consistent with the Western blot data shown in Figure 3(a). These findings provide evidence that 2-AP inhibits ER stress in the aorta of *apoE* ^−/−^ mice. 

## 4. Discussion 

The most important finding of this report is that oral feeding of the eIF-2*α* phosphorylation inhibitor 2-AP reduced atherosclerotic lesions in *apoE* ^−/−^ mice. This finding suggests that induction of eIF-2*α* phosphorylation is a causal mechanism for the development of atherosclerosis in *apoE* ^−/−^ mice.

Four eIF-2*α* kinases have been identified in mammalian cells, which each catalyze eIF-2*α* phosphorylation in response to various stressful cellular events [18, 19]. Specifically, ER stress activates PERK, heme deprivation activates heme-regulated inhibitor (HRI), virus infection activates double-stranded RNA-activated protein kinase (PKR) and amino acid deprivation activates general control nonderepressible-2 (GCN2) [19]. Previous studies from our laboratory demonstrated that treatment of mouse macrophages with E^−^/B48 lipoproteins induced phosphorylation of PERK but no other eIF-2*α* kinases and that overexpression of a nonphosphorylatable PERK mutant attenuated E^−^/B48 lipoprotein-induced eIF-2*α* phosphorylation and foam cell formation [7, 8]. These findings suggest that PERK is responsible for the increased eIF-2*α* phosphorylation observed in E^−^/B48 lipoprotein-treated macrophages. A high plasma level of E^−^/B48 lipoproteins is a hallmark feature of *apoE* ^−/−^ mice [10, 20]. Data from the present report demonstrated that 2-AP treatment reduced eIF-2*α* phosphorylation in the aorta *apoE* ^−/−^ mice. It is highly likely that plasma E^−^/B48 lipoproteins infiltrate into the arterial walls of *apoE* ^−/−^ mice, where they activate PERK and sequentially increase eIF-2*α* phosphorylation in vascular cells, including resident macrophages. 

A consequence of eIF-2*α* phosphorylation is upregulation of protein chaperons [19]. Indeed, phosphorylation of eIF-2*α* induced by E^−^/B48 lipoproteins has been associated with increased expression of calreticulin and GRP 78 [7]. Inhibition of eIF-2*α* by a nonphosphorylatable eIF-2*α* mutant or an 2-AP attenuated E^−^/B48 lipoprotein-induced expression of these protein chaperons [7]. Data from the present report showed that 2-AP treatment reduced the expression of GRP78. 

Reduction in global protein synthesis is another hallmark consequence of eIF-2*α* phosphorylation [4]. Under physiological conditions, inhibition of global protein synthesis and upregulation of protein chaperones reduces the accumulation of newly synthesized proteins in the ER, thereby relieving ER stress [21, 22]. However, constant phosphorylation of eIF-2*α* and inhibition of global protein synthesis might overpower normal cellular functions, causing pathological conditions [21, 22]. For example, we previously observed that E^−^/B48 lipoprotein-induced eIF-2*α* phosphorylation was associated with reduced global protein synthesis and reduced expression of lysosomal hydrolases, such as lysosomal acid lipase (LAL) [23]. In addition, we previously observed that inhibition of eIF-2*α* phosphorylation by 2-AP, or a nonphosphorylatable eIF-2*α* mutant, attenuated the reduced expression of LAL and inhibited foam cell formation induced by E^−^/B48 lipoproteins [7, 8]. LAL is the sole hydrolase responsible for cleavage of cholesteryl esters delivered to lysosomes [24, 25]. Taken together, our previous studies suggest that reduction in lysosomal hydrolases could be a fundamental mechanism by which E^−^/B48 lipoproteins trigger intracellular lipid/lipoprotein accumulation and transform macrophages into foam cells [23]. Correspondingly, the present report demonstrated that treatment of *apoE* ^−/−^ mice with 2-AP significantly elevated the protein level of LAL in the* apoE* ^−/−^ mouse aorta. It is possible that the E^−^/B48 lipoproteins deposited in the arterial wall induce eIF-2*α* phosphorylation, which downregulates lysosomal hydrolases, such as LAL, in the resident macrophages, and therefore reduces the degradation of E^−^/B48 lipoproteins leading to intralysosomal E^−^/B48 lipoprotein accumulation and resulting in foam cell formation and atherosclerosis development. This postulation is supported by the evidence showing that patients with a deficiency of LAL manifest both an accumulation of lipids in cells and develop premature atherosclerosis [25]. Further more, overexpression of LAL reduces atherosclerotic lesions in mice deficient in low-density lipoprotein receptor [26].

## 5. Conclusion

This report demonstrated that ER stress occurred in the aorta of *apoE* ^−/−^ mice, as reflected by increased phosphorylation of PERK and eIF-2*α* and by increased expression of chaperon GRP78. We also observed that inhibition of eIF-2*α* phosphorylation by 2-AP reduced atherosclerotic lesions in the aorta of *apoE* ^−/−^ mice. These observations suggest that induction of ER stress, that is, activation of the PERK-eIF2*α* signaling cascade, is a causal mechanism for development of atherosclerosis in *apoE* ^−/−^ mice. Such insights will inform novel strategies for using ER stress inhibitors such as 2-AP to prevent or treat atherosclerosis.

## Figures and Tables

**Table 1:** Mouse body weights and plasma lipids.

	Body weight (g)	Food intake (g/g BW/day)	Plasma cholesterol (mg/dL)	Plasma triglycerides (mg/dL)
Control	27.8 ± 0.5	0.25 ± 0.07	513 ± 29	146 ± 18
2-AP	26.9 ± 0.9	0.24 ± 0.03	530 ± 39	147 ± 17

Male *apoE* ^−/−^ mice at 6 weeks of age were gavage-fed 200 *μ*l water (control) or 200 mg/kg BW of 2-AP once every other day for 24 weeks. Body weight and food intake were monitored by weighing the animals and food weekly. Data presented are the final body weights at the end of the experiment and the average of food intake over 24 weeks. Blood samples were obtained from *apoE* ^−/−^ mice gavage-fed with 2-AP or water alone (control). Plasma total triglycerides and total cholesterol were determined as described in Materials and Methods. Values represent the mean SEM of 12 mice.

## Abbreviations

2-AP: 2-Aminopurine
Apo: Apolipoprotein
E^−^/B48 lipoprotein: Lipoproteins carrying apoB48 but lacking apoE
GRP78: Glucose-regulated protein 78
UPR: Unfolded protein response
LAL: Lysosomal acid lipase
ER: Endoplasmic reticulum
eIF-2*α*: Eukaryotic translation initiation factor 2*α*
PERK: RNA-dependent protein kinase-like ER kinase.
